# Perspectives on Social and Environmental Determinants of Oral Health

**DOI:** 10.3390/ijerph182413429

**Published:** 2021-12-20

**Authors:** Mauro Henrique Nogueira Guimarães de Abreu, Alex Junio Silva Cruz, Ana Cristina Borges-Oliveira, Renata de Castro Martins, Flávio de Freitas Mattos

**Affiliations:** Department of Community and Preventive Dentistry, School of Dentistry, Universidade Federal de Minas Gerais, Belo Horizonte 31270901, Brazil; aame0590@ufmg.br (A.J.S.C.); anacboliveira@ufmg.br (A.C.B.-O.); renatacm@ufmg.br (R.d.C.M.); fmattos@ufmg.br (F.d.F.M.)

**Keywords:** oral health, health behavior, delivery of health care, social determinants of health, environment and public health

## Abstract

Most oral conditions have a multifactorial etiology; that is, they are modulated by biological, social, economic, cultural, and environmental factors. A consistent body of evidence has demonstrated the great burden of dental caries and periodontal disease in individuals from low socioeconomic strata. Oral health habits and access to care are influenced by the social determinants of health. Hence, the delivery of health promotion strategies at the population level has shown a great impact on reducing the prevalence of oral diseases. More recently, a growing discussion about the relationship between the environment, climate change, and oral health has been set in place. Certainly, outlining plans to address oral health inequities is not an easy task. It will demand political will, comprehensive funding of health services, and initiatives to reduce inequalities. This paper sought to give a perspective about the role of social and physical environmental factors on oral health conditions while discussing how the manuscripts published in this Special Issue could increase our knowledge of the topic.

## 1. Introduction

The FDI World Dental Federation, one of the oldest dental associations in the world, stated a new concept for oral health as “multifaceted and includes the ability to speak, smile, smell, taste, touch, chew, swallow, and convey a range of emotions through facial expressions with confidence and without pain, discomfort, and disease of the craniofacial complex”. The driving determinants of oral health, which affect overall well-being, can be categorized in the individual’s physiological function, psychosocial function, and disease and condition status [1], and they also pointed out the determinants of oral health. Out of the five domains of what they have called “driving determinants”, at least two were the main focus of this Special Issue: social environment and physical environment. Health behaviors and access to care could be determined by the social environment [2,3,4,5].

Empirical evidence has been developed about the association between social determinants and a variety of oral health conditions [6,7,8,9,10,11,12], dental services utilization [13], and oral health behaviors [14,15]. On the other hand, few studies have focused on the relationship between the physical environment and oral health conditions. The association between challenges of the 21st century, such as a shortage of water, climate change, and oral health, do not have the same body of scientific evidence found about the association between social determinants and oral health. 

This paper aimed to describe and discuss some current evidence on the role of social and physical environmental factors on oral health conditions and pointed out how the manuscripts published in this Special Issue could contribute to our knowledge of the topic. Studies on the social and environmental determinants of health were identified through searches conducted on PubMed, Embase, and Google Scholar. Publications by the United Nations, the World Health Organization, and the World Dental Federation were also searched to organize this descriptive review. Manual searches were carried out using the keywords “oral health”, “determinants”, “environment”, “oral conditions”, “oral health behavior”, and “oral health services”. We focused on published systematic reviews about these topics. The literature search was completed in October 2021. 

## 2. Social Determinants of Oral Health

### 2.1. Oral Conditions

Dental caries remains the most prevalent oral disease. The prevalence of untreated dental caries in permanent dentition was estimated at 34.1% in 2015, impacting the age-standardized disability-adjusted life year [8]. A complex net of biological, behavioral, and social factors determines the disease, and systematic reviews (SR) have shown that poverty is also an important factor to be taken into account. Lower socioeconomic positions described by educational level, income, or occupation seems to increase caries experience in different age groups [6,7]. An SR carried out among children in Iran [16], the Middle East, and North Africa [17] reached similar conclusions. 

Severe chronic periodontitis affected 7.4% of the global population in 2015 [8]. Periodontal diseases are associated with tobacco consumption, comorbidities such as diabetes, and socioeconomic factors at both the individual and the population level. An SR approached the influence of a life-long individual-level socioeconomic position on adulthood periodontitis by evaluating seven longitudinal studies. It was found that, despite the limited number of papers and some methodological issues, a lower life-long socioeconomic position increased the risk of periodontitis in adulthood [18,19]. In this Special Issue, a cross-sectional study conducted with adults in London identified that periodontal disease was associated with individual and intersectional social characteristics, especially ethnicity and education [5]. Another cross-sectional study carried out among Indian adults also identified ethnicity as a relevant factor associated with periodontal disease [4]. Migration has been identified as impacting oral health. However, the way in which psychological and social factors affect migrants [20] and impact oral diseases needs to be better understood through both quantitative and qualitative research [20].

Head and neck cancer is the most common type of cancer worldwide, with projections of about half a million new cases yearly [21,22,23]. Previous research demonstrated that low income, low educational attainment, low socioeconomic position, and socio deprivation were positively associated with oral cancer [9,24]. In addition, a recent SR [25] reported that being from ethnic minority groups or being uninsured were related to either a delay in the diagnosis of oral cancer or a delay to start treatment. It was estimated that people affected by malignancies in the orofacial region had a substantial decrease in their oral health-related quality of life (OHRQoL), which impacted their ability to cope with daily activities [26]. More epidemiological studies evaluating the social determinants of those conditions are needed. 

Traumatic dental injuries (TDI) are a public health problem because of their high prevalence and also because a traumatized tooth may impact aesthetics, quality of life, and psychosocial behavior. It was estimated that over a billion people worldwide had TDI [27]. The global prevalence of TDI is 24.2% in primary dentition [28] and 15.2% in permanent dentition [27]. Until recently, evidence of an association between socioeconomic indicators and TDI was uncertain [29]. However, an overview of SR reported that some sociodemographic characteristics (younger age, male sex, and lower-income) were associated with a higher probability of being affected by TDI [10]. In the same study, the association between TDI and the educational level of caregivers remained unclear [10]. Further primary studies are required to fully understand how inequalities affect TDI.

Temporomandibular disorders (TMD) are the main cause of non-dental orofacial pain worldwide [12] and significantly affect people’s quality of life [30]. The prevalence of TMD among children and adolescents was found to vary between 7.3 and 30.4% [31]. The proportion of adults and elderly with TMD reached 31% [32]. However, the actual prevalence of the condition is still under debate due to variations in diagnostic criteria [33]. Despite that, there are some SR providing evidence that women [11,33] between 20–40 years of age [12] are more likely to develop TMD. The gender-related difference could be linked to biological, cultural, and social factors, but the pathways in which these factors predispose more women than men to TMD are not yet understood. Furthermore, the role of other sociodemographic indicators, such as ethnicity and socioeconomic status, on TMD prevalence is still controversial [22]. 

The broad knowledge of the social determinants of oral conditions could help decision-making by healthcare providers, the development of preventive programs by policymakers, and ultimately reduce oral health inequities [6,24,34,35].

### 2.2. Oral-Health-Related Behaviors

Despite improvements in prevention, oral diseases are still a significant population problem [14,36], associated with oral hygiene, tobacco use, diet, and stressors. Some psychosocial factors, such as ‘self-efficacy’, ‘intention’, ‘social influences’, ‘coping planning’, and ‘action planning’, have been associated with oral hygiene [15], and studies increasingly highlight the fact that positive health behavior is influenced by psychosocial factors. 

People with a higher sense of coherence (SOC) are better at managing stressful situations, problems, and promoting better general health. The SOC is a psychosocial determinant of people’s health behavior, which has been correlated to hygiene, dietary habits, and alcohol consumption [37]. An SR of nine articles aimed to analyze the empirical evidence on the association between oral health behaviors and SOC. The study identified that more favorable oral health behaviors were observed among those with higher SOC. This result suggested that SOC may be a determinant of oral-health-related behaviors, including frequency of toothbrushing, dental-care-seeking, and daily smoking habits. Mothers’ SOC could influence the oral health preventive practices of children [38]. Poursalehi et al. (2021) [36] performed an SR and meta-analysis to evaluate the effect of SOC on the oral health status of people in different age groups. The results showed that age, social support, education, working conditions, and living conditions in childhood could influence SOC. Gender did not show a significant effect on SOC. According to the authors, SOC appeared to be effective in predicting oral health behaviors.

Healthy habits such as daily toothbrushing, regular access to sources of fluoride, and moderate consumption of sugar are the most effective ways to prevent major oral diseases and to reduce health services costs [15]. According to Menegaz et al. (2018) [35], the strong social and behavioral character of oral diseases highlights the relevance of implementing educational interventions that encourage autonomy and change in health behaviors to promote prevention practices. 

Scheerman et al. (2016) [15] have carried out an SR and meta-analysis of 22 papers to identify the psychosocial determinants of oral hygiene behavior in people aged 9 to 19. Higher toothbrushing frequency among adolescents was associated with a higher ‘intention’, ‘social influences’, ‘self-efficacy’, ‘action planning’, and ‘coping planning’, suggesting that these factors are likely to be psychosocial determinants of tooth brushing. In the same study, the psychosocial variables: ‘locus of control’, ‘sense of coherence’, and ‘self-esteem’ were less likely to be associated with tooth brushing. The authors highlighted the importance of psychosocial factors as determinants of oral hygiene habits among pre-adolescents and adolescents. The results of an SR developed by Calderon et al. (2014) [14] showed that ethnicity, race, and gender could affect the oral health behavior of adolescents. The results of an SR developed by Menegaz et al. (2018) [35] concluded that educational interventions promote changes in oral-health-related behaviors (daily tooth brushing, regular contact with fluoride sources, and controlled consumption of sugar), prevent major oral diseases, and reduce costs for health services and society.

It is very important that effective programs improve oral-health-related behavioral habits in different age groups. Literature indicates the need to develop research on other factors that affect oral health behavior. Future intervention trials should consider a range of psychological factors that have not been fully studied, such as ‘self-determination’, ‘anticipated repentance’, ‘action control’, and ‘self-identity’.

### 2.3. Dental Services Utilization

The access and use of dental services are relevant information to be studied in different populations. Ethnics minorities or immigrants and those from low socioeconomic groups showed lower dental services utilization globally [39]. A higher-income was consistently associated with children’s use of dental services. Among adults, 50% of the observational studies included in an SR have identified more education as a factor that increases dental service utilization [40]. In the older population, the rate of annual dental visits among those with a higher income and higher socioeconomic status is higher than among those with a lower income [41,42]. 

An SR showed that regular or preventive dental services utilization differs greatly across the globe. In countries with a higher human development index (HDI), more individuals utilize services. The utilization is also highly unequally distributed between different groups within countries. Individuals with less-supportive family structures, poor health literacy, poor general and oral health, edentulous or with severe tooth loss, and younger children show a lower utilization. Neither utilization nor its differences between groups have changed significantly with time [13]. While the burden of oral diseases is heavier on more socially vulnerable populations, access to dental services is better for those with higher socioeconomic conditions [40]. There is a positive association between dental insurance and dental visits [43]. Dentally insured adults have more regular access to dental care than the uninsured. This inequity situation increases the gap between the rich and poor. In our Special Issue, organizational and human resources factors were associated with access to dental prostheses in Brazil. Public dental offices with better organizational support and improved work incentives provided more dental prostheses to their patients. The reduction of inequalities in primary oral health care access should be set by policymakers [2]. 

Apart from identifying oral health determinants, it is urgent to overcome inequalities to promote health and to impact different morbidities, including oral conditions. An SR [44] evaluated intervention programs developed to reduce inequality in dental caries prevalence among children. The studied interventions included health promotion/preventive initiatives, topical fluorides, and water fluoridation to reduce caries among children of different socioeconomic groups. Comparison groups included children with an alternative or no intervention. The findings suggested that broader population interventions such as water fluoridation are more likely to reduce inequalities in children’s caries than interventions targeted at specific populations. 

Studies of interventions to reduce socioeconomic inequalities [45] in dental service utilization by adults are limited to those involving pregnant women and parents organizing care for their children. They have been mostly targeted at individual behavior, rather than community or structural factors and involved participants at the lower end of the socioeconomic status spectrum. Evidence involving participants across the whole social gradient is limited. 

There is a lack of research on interventions that aim to reduce socioeconomic inequalities in adult dental visits and on interventions that target community or structural causes of these inequalities. In our Special Issue, a trial identified how an integrated oral healthcare intervention for pregnant women impacted health outcomes. This study revealed that despite socioeconomic and behavioral health determining factors, multi-professional health actions during prenatal care could contribute to positive pregnancy outcomes and oral health [3]. Health policies and services availability influence inequality in services provision, while individual, social, cultural, and economic determinants affect inequality in dental services utilization [42].

## 3. And What about the Environmental Determinants of Health?

### 3.1. Health Determinants

Published in 1991 [46,47], and revised in 2021, the Dahlgren and Whitehead model has been widely used worldwide. It focuses on the determinants of health, rather than on the causes of the diseases, to enable people and non-medical professionals to act to improve health. The focus on the determinants of health allows the development of more comprehensive strategies in which actions can be planned and implemented without the risk of fragmentation induced by focusing on the aetiology of diseases. Specialized uncoordinated actions to treat and prevent different diseases have very limited impact in reducing risk factors or determinants of health outside their immediate field when compared with comprehensive strategies for the determinants of health. The model is a pathway to inequalities in health that proposes four main influences on the determinants of health: differential power and resources; differential exposure; differential vulnerability; and differential consequences of being sick or healthy.

Many of the social determinants for good health, such as livelihoods, equality, access to health care, and social support structures, are being undermined by climate change. Climate-sensitive health risks are particularly felt by the most vulnerable: women, children, ethnic minorities, poor communities, migrants or displaced persons, elderly populations, and those with underlying health conditions [48]. The marked social gradient in health within and between countries, and its associated inequities, are caused by the unequal distribution of power, income, goods and services, access to health care, schools and education, conditions of work and leisure, housing, communities, and towns. This unequal distribution of health-damaging experiences is the result of the combination of poor social policies and programmes and unfair economic arrangements. The structural determinants and conditions of daily life constitute the social determinants of health and are responsible for a major part of health inequities [49].

### 3.2. Environmental Health Determinants

Environment is one of the determinants of health. Both social environment (social support and social networks, social deprivation, income inequality, racial discrimination, social cohesion, and social capital) and built environment (human-made or modified surroundings) have been systematically reviewed to understand their impact on health outcomes [50,51]. Dental caries, fluorosis, and their association with fluoridated water [52,53] are probably the most frequently studied environmentally determined oral health conditions. As one of the most prevalent chronic diseases, dental caries affect some 70% of children among disadvantaged families globally. The disease affects more ethnic minorities, people living in rural areas, and socially disadvantaged children. A systematic review has shown that fluoride use reduces the incidence of caries in lower socioeconomic areas. Children in higher socioeconomic positions may already have good oral-health-related behaviours, better access to dental services, and more access to fluoride toothpaste when compared to children in lower socioeconomic positions. Thus, the provision of fluoridated water and the use of fluoride-containing products is more likely to be useful among those in lower socioeconomic positions [44].

Climate change impacts health in many ways, leading to death and illness from increasingly frequent extreme weather events, such as heatwaves, storms, and floods. It disrupts food systems, increases zoonoses, food, water, and vector-borne diseases, and generates adverse mental health outcomes. A half-century of progress in development, global health, and poverty reduction is threatened by climate change, for it widens health inequalities. It threatens universal health coverage by increasing the burden of diseases and by reinforcing barriers to health services access. Some 12% of the world’s population spend at least 10% of their household budget to pay for health care. Health shocks and stresses caused by climate change push around 100 million people into poverty every year [48]. 

The effects of environmental changes on health will affect most populations in the next decades. Their management will require inputs and coordination from all sectors of government, a collaboration between countries, academic institutions, and disciplines. Local communities must be involved in monitoring, discussing, and advocating and require assistance with their process of adaptation. A multidisciplinary approach to reduce the adverse health effects of environmental changes requires three levels of action. First, policies must reduce carbon emissions and increase carbon biosequestration to eventually stabilize temperatures. Second, political and social action should be taken on the events that link climate change to disease. Third, appropriate public health systems should be put into place to deal with the adverse outcomes of climate change [54].

The multiple health impacts of climate change include an increase in infectious diseases, respiratory disorders, heat-related morbidity and mortality, undernutrition due to food insecurity, and increased sociopolitical tension and conflicts [51]. These effects are frequently unequal and impact disproportionately both populations who have and have not contributed to the problem. Climate change interacts with existing social and economic inequalities and exacerbates gaps within and between countries [55]. A recent overview of SR has summed up the indirect and direct interdependence of environmental well-being and human well-being. Temperature and humidity rises have been associated with infectious diseases, mortality, and adverse respiratory, cardiovascular, and neurological outcomes. Others less-frequently studied, but with consistent associations, included those between climate impacts and increased use of healthcare services, adverse mental health outcomes, adverse nutritional outcomes, and adverse occupational health outcomes [51].

### 3.3. Peoples’ Response and Sustainability

A recent SR has described evidence on the effects of climate change adaptation responses on health outcomes in low- and middle-income countries as disparate and limited [56]. The topic lacked further quantitative data, existing evaluation timelines were described as typically short, and there were no studies reporting health outcomes in periods longer than 12 months. Papers approaching responses to extreme weather events were frequent, whilst responses to gradual climate changes have been scarcely studied. The effect of climate change adaptation responses on infectious diseases, food security, and indicators of household access to drinking water, sanitation, and hygiene (WASH) has been studied. However, evidence that these adaptation responses improved WASH indicators and food security is limited [57]. It is unequivocal that climate change affects human health. However, it remains challenging to accurately estimate the scale and impact of many climate-sensitive health outcomes.

Sustainable development is defined as the development that meets the needs of the present without compromising the ability of future generations to meet their own needs. Economic growth, social inclusion, and environmental protection are the three main different pillars of sustainable development. The United Nations has set and monitored 17 sustainable development goals for 2030. They are: to end poverty in all its forms everywhere; to end hunger, achieve food security and improved nutrition, and promote sustainable agriculture; to ensure healthy lives and promote well-being for all at all ages; to ensure inclusive and equitable quality education and promote lifelong learning opportunities for all; to achieve gender equality and empower all women and girls; to ensure availability and sustainable management of water and sanitation for all; to ensure access to affordable, reliable, sustainable, and modern energy for all; to promote sustained, inclusive and sustainable economic growth, full and productive employment and decent work for all; to build resilient infrastructure, promote inclusive and sustainable industrialization and foster innovation; to reduce inequality within and among countries; to make cities and human settlements inclusive, safe, resilient and sustainable; to ensure sustainable consumption and production patterns; to take urgent action to combat climate change and its impacts; to conserve and sustainably use the oceans, seas and marine resources for sustainable development; to protect, restore and promote sustainable use of terrestrial ecosystems, sustainably manage forests, combat desertification, and halt and reverse land degradation and halt biodiversity loss; to promote peaceful and inclusive societies for sustainable development, provide access to justice for all and build effective, accountable and inclusive institutions at all levels; and to strengthen the means of implementation and revitalize the Global Partnership for Sustainable Development [58].

## 4. Final Comments

This paper approached the social and physical environmental determinants of oral health and critically discussed them to identify and explain their relationship. Further scientific and political actions are needed to reduce inequalities and to promote health. In the 21st century, the impact of the physical environment on some health outcomes has already been identified. However, it should be emphasized that their same impact on oral health outcomes is not fully understood. It is urgent that researchers and policymakers dedicate resources to the subject.

## Data Availability

The data presented in this study are available on request from the corresponding author.
